# Development of Curcumin/ADP-Loaded Gelatin Methacrylate Hydrogel for Enhanced Wound Healing with Hemostatic, Anti-Inflammatory, and Antibacterial Properties

**DOI:** 10.3390/gels12060456

**Published:** 2026-05-22

**Authors:** Awn Abbas, Nanxin Li, Sameera Naseer, Lian Chen, Xiaoyang Ai, Yixing Chen, Chongde Gu, Hualin Fu

**Affiliations:** Innovative Engineering Research Center of Veterinary Pharmaceutics, Department of Pharmacy, College of Veterinary Medicine, Sichuan Agricultural University, Chengdu 611130, China; awni1115@gmail.com (A.A.); linanxinao@163.com (N.L.); sameeranaseer38@gmail.com (S.N.); 15908499457@163.com (L.C.); a1600063002@163.com (X.A.); chenyixing@stu.sicau.edu.cn (Y.C.); a1234567896016@163.com (C.G.)

**Keywords:** wound healing, Gelatin Methacrylate anhydride, hydrogel, curcumin, adenosine 5-diphosphate

## Abstract

Gelatin methacrylate (GelMA) hydrogels are promising carriers for bioactive agents like curcumin (Cur) and adenosine diphosphate (ADP) in wound healing. However, existing GelMA-based systems fail to achieve both rapid hemostasis and sustained anti-inflammatory effects. In this study, we developed a Cur/ADP GelMA hydrogel, and evaluated its anti-inflammatory, regenerative, hemostatic, and biocompatible properties. Proton nuclear magnetic resonance (^1^H-NMR) analysis showed that a 65% degree of substitution of GelMA is optimal for wound dressings. Scanning electron microscopy revealed a uniform pore size, aiding inflammatory exudate removal. The Cur/ADP GelMA hydrogel exhibited strong adhesion, stability, and antibacterial activity, reducing *E. coli* and *S. aureus* proliferation by 85% and 72%, respectively. Hemostatic effects were observed, with blood loss reduced to 238 ± 23 mg compared to 559 ± 18 mg in the untreated group. The ELISA results showed reduced pro-inflammatory cytokines (TNF-α, IL-1β, IL-6) and increased IL-10. In vivo studies demonstrated 98% wound closure by day 14, enhanced granulation tissue formation, and a 70% thicker epidermis compared to controls. Mechanistically, ADP accelerates platelet activation and clot formation, while Cur modulates the inflammatory microenvironment, enabling synergistic hemostasis and immune regulation, thus promoting accelerated wound healing.

## 1. Introduction

Traumatic wounds compromise tissue integrity, disrupt the skin barrier, and pose a major global health challenge, particularly when accompanied by bacterial infection and chronic inflammation [1]. Wound healing is a highly coordinated biological process involving four interconnected phases, namely, hemostasis, inflammation, proliferation, and remodeling, which collectively restore tissue integrity [2]. Nevertheless, uncontrolled traumatic injuries, especially those resulting from road accidents, military conflicts, and natural disasters, constitute critical medical emergencies and remain a leading cause of preventable mortality. Most trauma-related deaths occur within 60 min of injury; therefore, early bleeding control is important for survival [3]. Exposure of wounds to the external environment often leads to bacterial colonization, which significantly impairs healing by elevating oxidative stress and sustaining inflammatory responses. Traditional wound management strategies rely on conventional dressings (bandages, gauze, and foam) and systemic antibiotics, either as monotherapy or in combination. However, the widespread excessive use of antibiotics has led to the rise of drug-resistant pathogens, rigorously limiting therapeutic efficiency and necessitating alternative antimicrobial strategies [4]. Among the available wound dressing materials, hydrogels have emerged as one of the most favorable options due to their ability to maintain a moist wound environment, absorb exudate, provide cooling effects, and serve as drug delivery systems [5,6].

Hydrogels are biocompatible three-dimensional networks with physicochemical properties similar to those of the extracellular matrix, which makes them particularly attractive for use as tissue engineering scaffolds [7,8]. The tunability of hydrogelation in bioinspired short peptides offers significant potential for developing versatile, responsive materials, which can be tailored for various biomedical applications, including tissue engineering and wound healing [9]. Hydrogels can be prepared from natural polymers such as chitosan and gelatin, with gelatin often being preferred because of its excellent biocompatibility, biodegradability, and close resemblance to the natural extracellular matrix [10]. Gelatin methacrylate (GelMA) is synthesized by reacting gelatin with methacrylic anhydride and is widely used in wound healing due to its biocompatibility and ability to support cell adhesion [11]. Additionally, its degree of substitution (DS), defined as the ratio of amino groups substituted by methacrylate, can be easily adjusted according to specific requirements [12].

The incorporation of bioactive compounds into the hydrogel matrices is a favorable approach to tackle the issue of antibiotic resistance. Curcumin is a polyphenolic bioactive compound extracted from the rhizome of *Curcuma longa* plant [13], and possesses antioxidant, antibacterial, anti-inflammatory, and healing properties [14]. It promotes wound healing by scavenging free radicals; enhancing antioxidant enzyme expression; suppressing pro-inflammatory cytokines such as IL-6, TNF-α, and IL-1β; and stimulating granulation tissue formation, angiogenesis, and collagen synthesis [8,15]. Additionally, it has shown efficacy against wound pathogens like *S. aureus* and *E. coli*, which renders it as a potential antibiotic substitute in the management of infected wounds. In a recent study, Wei et al. developed a GelMA/alginate-tannic acid hydrogel with strong antioxidant and antibacterial properties that accelerated full-thickness wound closure by modulating the inflammatory response and promoting granulation tissue formation [16].

In addition to antimicrobial and anti-inflammatory activities, effective hemostasis is a critical requirement in traumatic wound management. In this context, adenosine diphosphate (ADP), a natural platelet agonist, plays an important role by promoting platelet aggregation at the injury site through activation of the G-protein-coupled receptors P2Y1 and P2Y12 [17,18]. In a study, Zhou et al. reported that a self-assembled J-1-ADP hydrogel, generated from the antimicrobial peptide jelleine-1 and ADP sodium solution, exhibited potent antibacterial and hemostatic activities, as demonstrated by whole-blood coagulation, plasma coagulation, platelet activation, and platelet adhesion assays [19]. In a similar study, Hong et al. designed a photo-crosslinkable adhesive hemostatic hydrogel that forms a bond to wet and dynamic tissues upon exposure to UV light to seal high-pressure arterial and cardiac wounds [3]. Although considerable progress has been made in wound care, there is still no ideal clinical intervention that can effectively and simultaneously overcome the four principal challenges of wound healing: hemostasis, bacterial infection, oxidative stress, and chronic inflammation. Current treatment approaches often target these obstacles individually, thereby increasing therapeutic complexity and reducing overall treatment efficiency. Consequently, the development of a single multifunctional wound dressing that integrates rapid hemostatic function with antibacterial, antioxidant, and anti-inflammatory properties would represent a significant advance in the field.

To address this unmet need, we developed a novel multifunctional Cur/ADP GelMA hydrogel that combines the antibacterial, antioxidant, and anti-inflammatory activities of curcumin with the hemostatic and immunomodulatory functions of ADP in a biocompatible GelMA matrix and demonstrated, through in vitro and in vivo evaluation, its strong potential as an advanced dressing for traumatic wound management.

## 2. Results and Discussion

### 2.1. Determination of Degree of Substitution (DS)

**Figure 1:**
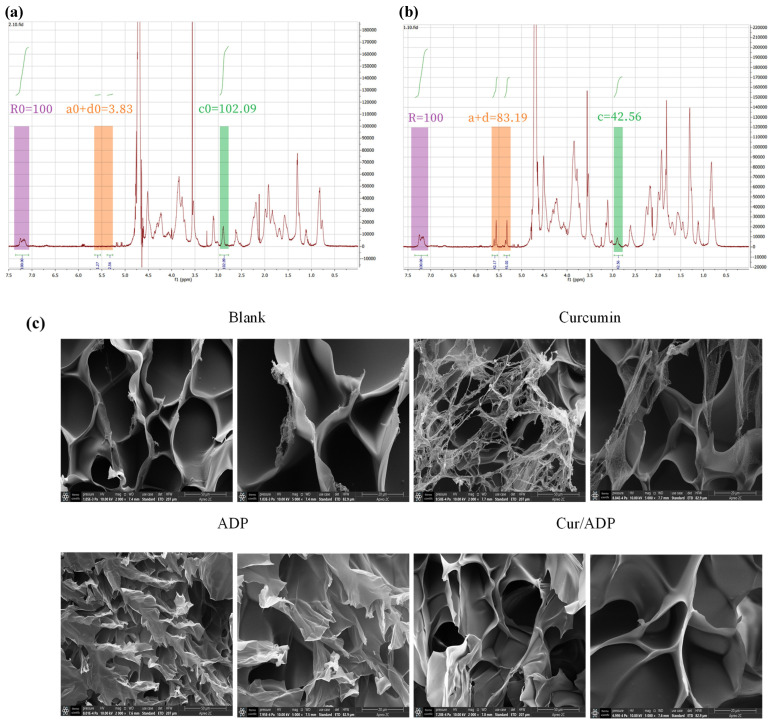

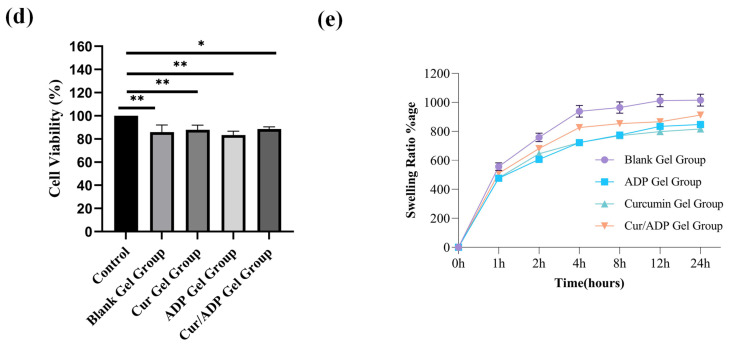
Characterization of GelMA hydrogels. (**a**) ^1^H-NMR spectrum of gelatin raw material used to prepare GelMA. (**b**) ^1^H-NMR spectrum of GelMA product, confirming successful methacryloyl functionalization. (**c**) SEM images of all hydrogel groups at 50 µm and 20 µm resolutions. Blank GelMA shows a regular, interconnected porous structure, while curcumin-loaded and ADP-loaded GelMA exhibit more compact and irregular pores. The Cur/ADP-loaded GelMA maintains a uniform, interconnected porous network. SEM images were acquired at 2000× magnification with 50 µm scale bars and 5000× magnification with 20 µm scale bars. (**d**) Cell viability of all hydrogel groups, showing no significant cytotoxicity with viability above 80% in all formulations. (**e**) Swelling kinetics of GelMA hydrogels. Blank GelMA exhibited the highest swelling, followed by Cur/ADP-loaded GelMA, ADP-loaded GelMA, and curcumin-loaded GelMA. * *p* < 0.05; ** *p* < 0.01.

^1^H-NMR spectra confirmed the successful synthesis of GelMA. Compared with raw gelatin, GelMA showed characteristic methacryloyl vinyl proton signals in the 5.4–5.7 ppm region, confirming the introduction of photo-crosslinkable methacryloyl groups. Meanwhile, the signal around 2.7–3.2 ppm was attributed to residual lysine methylene/free amino protons rather than succinimide functionalities, because succinimide-containing reagents were not used during GelMA synthesis. The calculated DS value of GelMA was 65.09%, indicating moderate methacryloyl substitution of gelatin. This level of substitution is suitable for hydrogel formation because it provides sufficient photo-crosslinkable groups while preserving the intrinsic biocompatibility of gelatin. This level of substitution aligns with previous studies, which highlight the importance of precise functionalization in hydrogels to enhance their performance in biomedical applications [20]. SEM analysis of GelMA hydrogel morphology was performed to comprehensively assess its structural integrity, surface characteristics, and porosity, which are critical for understanding its performance in biomedical applications [21]. Figure 1c shows the porous network present in all hydrogel groups. Blank GelMA exhibited a regular, interconnected structure, while curcumin- and ADP-loaded GelMA had more compact, irregular pores. The Cur/ADP-loaded GelMA maintained a uniform, interconnected porous structure, suggesting that dual-drug incorporation preserved the network’s integrity. These changes in pore structure upon drug incorporation are consistent with reports by [22], who demonstrated that drug loading can significantly affect the morphology of GelMA hydrogels. A cell viability assay was performed on the blank and drug-loaded GelMA hydrogel to evaluate its biocompatibility and to determine its potential for supporting cellular growth and function in tissue engineering applications [23]. The cell viability (Figure 1d) remained above 80% in all hydrogel groups, with no cytotoxicity observed, confirming the biocompatibility of GelMA and its drug-loaded formulations, which is in line with prior research on GelMA’s suitability for tissue engineering application [24]. A swelling behavior assay was performed on the GelMA hydrogel to investigate its water absorption capacity and structural stability, which are crucial for its functionality in dynamic physiological environments [25]. The swelling behavior (Figure 1e) showed that blank GelMA had the highest swelling ratio, followed by Cur/ADP-loaded, ADP-loaded, and curcumin-loaded GelMA. Drug loading reduced swelling, with the Cur/ADP-loaded GelMA showing higher swelling than the single-drug formulations but still lower than blank GelMA. The swelling trend was as follows: Blank GelMA > Cur/ADP-loaded GelMA > ADP-loaded GelMA > curcumin-loaded GelMA. This trend mirrors findings from [26], suggesting that drug incorporation leads to a decrease in swelling due to increased crosslinking.

### 2.2. Sol–Gel Transition, Drug Complexation, Release Behavior, and Tissue Adhesion of Cur/ADP-Loaded GelMA Hydrogel

**Figure 2:**
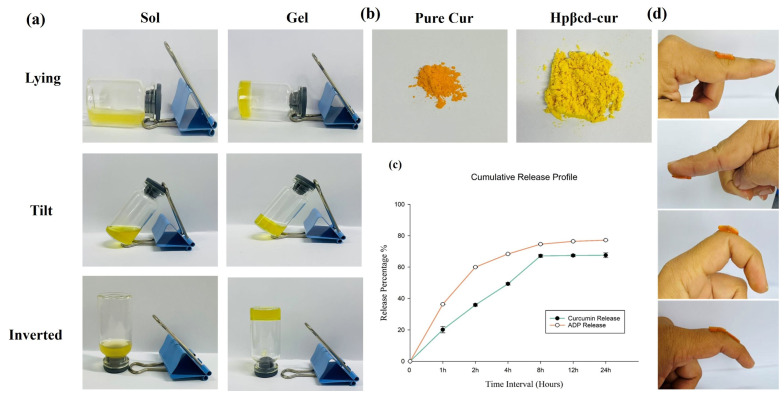
Characterization and release profile of Cur/ADP-loaded GelMA hydrogel. (**a**) Sol–gel transition: The sol form of Cur/ADP-loaded GelMA in lying, tilted, and inverted positions before UV exposure (sol); gelation of the hydrogel after UV exposure in the same positions (gel). (**b**) Physical appearance of pure curcumin and HPβCD-cur inclusion complex. (**c**) In vitro cumulative release profiles of curcumin and ADP from the gel over time. (**d**) Photograph of Cur/ADP-loaded hydrogel applied to a human knuckle, demonstrating its adhesive properties.

To check the integrity of GelMA, the sol–gel transition was carried out and as shown in Figure 2a, the Cur/ADP-loaded GelMA precursor remained in a flowable sol state before UV irradiation, as evidenced by its mobility in the lying, tilted, and inverted positions. After UV exposure, the formulation rapidly transformed into a stable gel and retained its shape without obvious flow under the same positional changes, confirming successful photo-crosslinking and effective sol–gel transition. This rapid transformation is consistent with prior studies demonstrating the efficient sol–gel transition of GelMA hydrogels, which is crucial for in situ gelation in wound healing applications [27]. The physical appearance of pure curcumin and the HPβCD-cur inclusion complex is presented in Figure 2b. Compared with pure curcumin, the HPβCD-cur complex showed a visibly altered powder appearance, indicating successful incorporation of curcumin into the HPβCD host system. This change in physical form further supports the improved dispersion of curcumin after complexation. The in vitro cumulative release profiles showed sustained release of both bioactive agents from the hydrogel matrix (Figure 2c). ADP exhibited a relatively faster release rate during the initial period, reaching about 36% at 1 h and 60% at 2 h, followed by a slower increase to nearly 78% at 24 h. In comparison, curcumin was released more gradually, with cumulative release reaching about 20% at 1 h, 36% at 2 h, 49% at 4 h, and approximately 68% at 24 h. The swelling of the GelMA hydrogel facilitates the diffusion of curcumin and ADP, promoting their release from the matrix. However, as the swelling is reduced in drug-loaded formulations, the diffusion of the drugs becomes more controlled, leading to a slower, sustained release. This controlled release is crucial for maintaining a prolonged therapeutic effect at the wound site.

These results indicate that the hydrogel provided a controlled and prolonged release pattern, with ADP being released earlier than curcumin. This sustained release pattern is indicative of the hydrogel’s potential as a controlled drug delivery system. As shown in Figure 2d, the Cur/ADP-loaded GelMA hydrogel adhered well to the human knuckle and remained attached during finger bending and positional changes. This result demonstrates that the hydrogel possessed good tissue adhesion and mechanical adaptability, which are important for maintaining intimate wound contact on mobile skin surfaces.

### 2.3. In Vitro and In Vivo Antibacterial Activity of Cur/ADP GelMA Hydrogels

**Figure 3:**
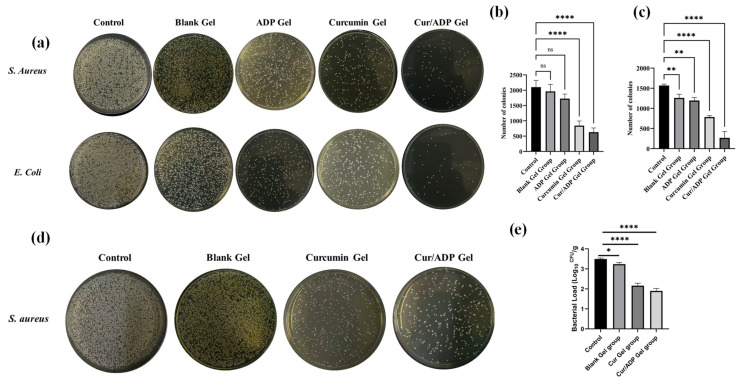
In vitro and in vivo antibacterial activity of drug-loaded GelMA hydrogels. (**a**) Representative images showing the growth of *S. aureus* and *E. coli* following treatment with different GelMA hydrogel formulations. (**b**) In vitro survival rate of *S. aureus* colonies after treatment with the respective hydrogel groups. (**c**) In vitro survival rate of *E. coli* colonies after treatment with the respective hydrogel groups. (**d**) In vivo images of *S. aureus* growth following treatment with different hydrogel formulations. (**e**) Bacterial load (Log_10_ CFU/g) of *S. aureus* in vivo after treatment with different hydrogel groups. * *p* < 0.05; ** *p* < 0.01; **** *p* < 0.0001; ns = not statistically significant.

The antibacterial activity of different GelMA formulations against *S. aureus* and *E. coli* is shown in Figure 3a–c. Plate images revealed dense bacterial colonies in the control and blank gel groups for both strains, indicating that the unloaded GelMA hydrogel had little intrinsic antibacterial activity. In contrast, drug-loaded hydrogels reduced colony formation to different extents. For *S. aureus*, the ADP gel showed no significant reduction compared with the blank gel, whereas the curcumin gel markedly decreased the number of colonies. The Cur/ADP gel exhibited the strongest antibacterial effect, producing the lowest colony count among all groups (Figure 3a,b). A similar trend was observed against *E. coli* (Figure 3a,c). Both the ADP gel and curcumin gel significantly reduced bacterial colonies compared with the blank gel, while the Cur/ADP gel achieved the greatest inhibition and showed the lowest survival of *E. coli* colonies. The in vivo antibacterial activity against *S. aureus* further confirmed the superiority of the drug-loaded formulations (Figure 3d,e). Compared with the control and blank gel groups, both curcumin-loaded hydrogels markedly reduced bacterial growth in infected tissue. The quantitative analysis of bacterial load showed that the control and blank gel groups maintained the highest bacterial burden, whereas the Cur/HPβCD gel significantly decreased the bacterial count. Notably, the Cur/ADP gel produced the lowest bacterial load among all groups, demonstrating the most effective in vivo antibacterial performance. These results indicate that the incorporation of curcumin substantially enhanced the antibacterial activity of GelMA hydrogel, and dual loading with curcumin and ADP further improved bacterial inhibition both in vitro and in vivo. The incorporation of curcumin, a potent antioxidant and antibacterial agent, significantly reduced bacterial growth, corroborating findings from previous studies on curcumin-loaded hydrogels [28]. A plant-derived curcumin hydrogel shows promising wound healing efficacy in mouse models, highlighting its potential as a biocompatible therapeutic approach alongside advances in crop protection and smart agriculture [29].

### 2.4. Hemostatic Performance and Hemocompatibility of the Hydrogel

**Figure 4:**
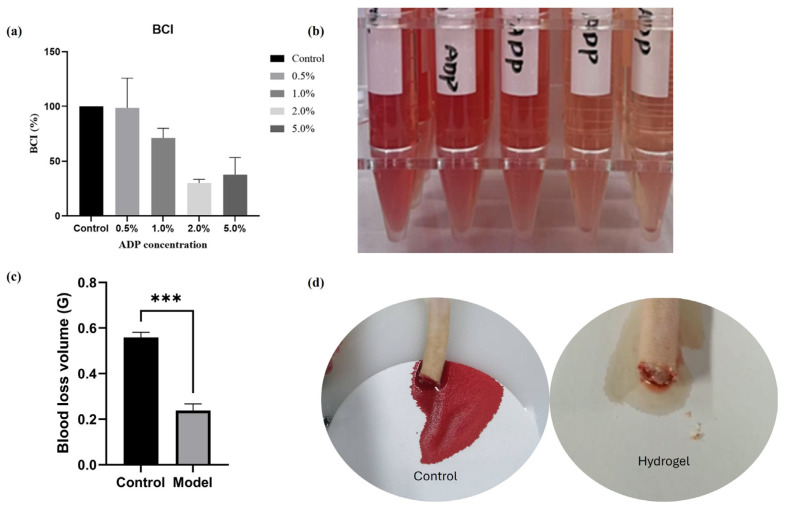
Evaluation of hemostatic properties of hydrogels. (**a**) Blood Clotting Index (BCI) of different ADP-loaded hydrogel concentrations. (**b**) Hemolysis images showing red blood cell sediments after treatment with various concentrations of ADP-loaded hydrogels. (**c**) Quantitative analysis of blood loss volume following hydrogel treatment in an in vivo rat tail amputation model. (**d**) Digital images showing blood loss after treatment with control and hydrogel formulations in the rat tail amputation model. All data are expressed as mean ± SD (*n* = 3). Statistical analysis was performed using one-way ANOVA. *** *p* < 0.001.

The Blood Clotting Index was calculated to evaluate the hydrogel’s hemostatic potential, with the results shown in Figure 4a. Compared with the control group, the incorporation of ADP reduced the BCI value, indicating enhanced blood clotting ability. The 0.5% ADP group showed little change relative to the control, whereas the 1.0% ADP group showed a clear decrease in BCI. The lowest BCI was observed in the 2.0% ADP group, suggesting the strongest procoagulant effect at this concentration. Although the 5.0% ADP group also exhibited a reduced BCI compared with the control, its clotting performance was weaker than that of the 2.0% group. These results indicate that ADP improved the coagulation capacity of the hydrogel in a concentration-dependent manner, with 2.0% showing the most effective clot-promoting activity. Hemolysis analysis further demonstrated the acceptable blood compatibility of the ADP-loaded hydrogels (Figure 4b). As the ADP concentration increased, the supernatant became progressively clearer and a greater amount of red blood cell sediment was observed at the bottom of the tubes, indicating reduced hemolysis and better erythrocyte preservation after treatment. The in vivo rat tail amputation model further confirmed the hemostatic efficacy of the hydrogel (Figure 4c,d). Quantitative analysis showed that the hydrogel treatment significantly reduced blood loss compared with the untreated control group. The blood loss in the hydrogel-treated group was less than half that of the control, demonstrating a marked hemostatic effect. Consistently, digital images showed severe bleeding in the control group, whereas hydrogel application rapidly limited bleeding at the wound site. The hemostatic performance of the hydrogel was impressive, with a reduced Blood Clotting Index (BCI) and significantly lower blood loss in the rat tail amputation model. This is consistent with earlier work, which indicated that ADP enhances platelet aggregation and accelerates wound closure [19].

### 2.5. In Vivo Wound Healing and Tissue Regeneration Promoted by Cur/ADP-Loaded GelMA Hydrogel

**Figure 5:**
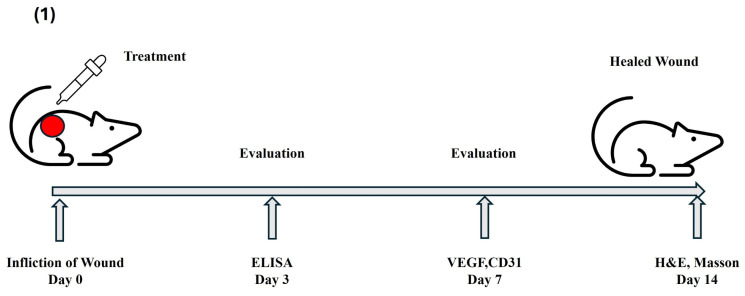

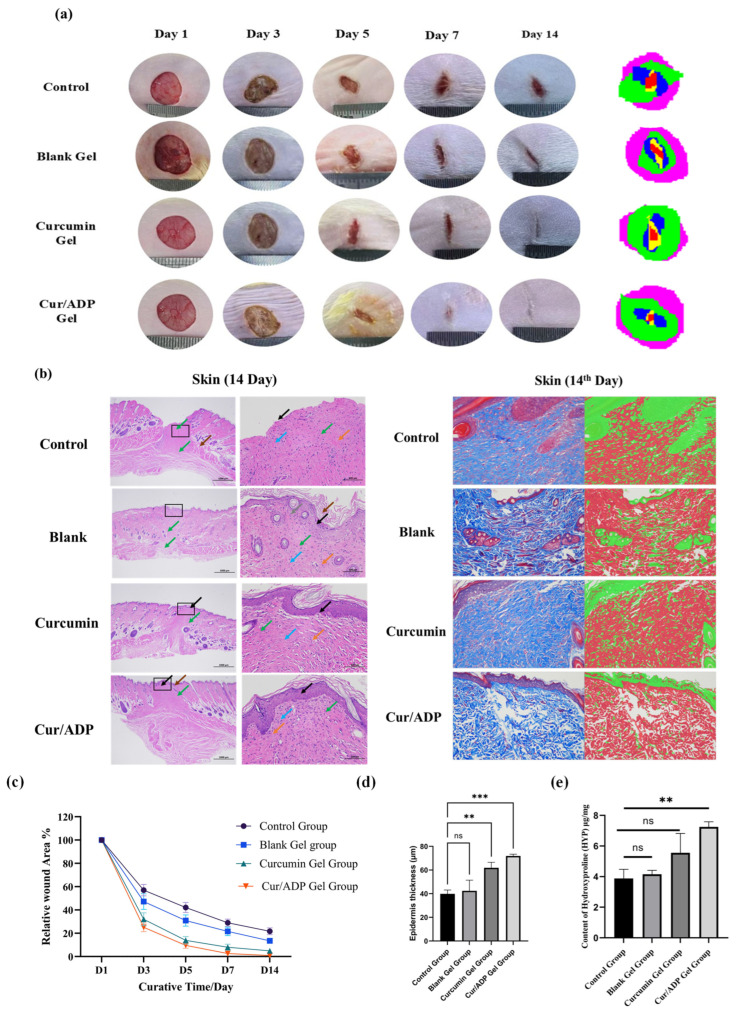
In vivo wound healing analysis of hydrogel treatment. (**1**) Schematic representation of the hydrogel treatment procedure for wound healing. (**a**) Day-by-day progression of wound healing for each treatment group. (**b**) H&E staining and Masson’s trichrome staining images of the wound area on day 14, showing histological differences in tissue regeneration across treatment groups. (**c**) Quantitative analysis of wound contraction and relative wound healing percentage over the 14-day period *(n* = 5). (**d**) Statistical analysis of epidermal thickness across treatment groups on day 14. (**e**) Quantification of hydroxyproline (HYP) content (*n* = 3) in the wound area. All data are presented as means ± SD. Statistical significance was determined by one-way ANOVA with Tukey’s multiple comparisons test. ** *p* < 0.01; *** *p* < 0.001; ns = not statistically significant.

The wound-healing procedure is illustrated schematically in Figure 5-1, showing hydrogel treatment immediately after wound induction on day 0, followed by sequential evaluation of inflammation, angiogenesis, and tissue remodeling during the healing process. Macroscopic observation showed that all wounds gradually contracted over time, but the healing rate differed markedly among groups (Figure 5a). The Cur/ADP gel group exhibited the fastest wound closure throughout the observation period, with a marked reduction in wound area from day 3 onward and near-complete healing by day 14. The curcumin gel group also accelerated wound repair compared with the control and blank gel groups, whereas the control group retained the largest residual wound area during the entire experimental period. Quantitative analysis of wound contraction further confirmed these observations (Figure 5c). The relative wound area decreased progressively in all groups, but the decline was most pronounced in the Cur/ADP gel group, followed by the curcumin gel group. By day 14, the Cur/ADP gel-treated wounds showed the smallest remaining wound area, indicating the highest wound-healing efficiency. In contrast, the control group showed the slowest closure, while the blank gel group produced only a moderate improvement. The histological evaluation on day 14 revealed clear differences in tissue regeneration among the treatment groups (Figure 5b). H&E staining showed that the Cur/ADP gel group had a more continuous and intact epidermal layer, better re-epithelialization, and more complete restoration of skin architecture than the other groups. Skin appendages and newly formed tissue structures were more evident in the Cur/ADP gel group, whereas the control and blank gel groups still showed incomplete epithelial coverage and less organized tissue repair. The quantitative analysis demonstrated that epidermal thickness was significantly greater in the curcumin gel and Cur/ADP gel groups than in the control and blank gel groups, with the Cur/ADP gel group showing the highest value (Figure 5d). Masson’s trichrome staining further showed enhanced collagen deposition and extracellular matrix remodeling in the drug-loaded groups (Figure 5b). Collagen fibers in the Cur/ADP gel group appeared denser, more abundant, and better organized than those in the other groups, indicating more advanced dermal reconstruction. This finding was supported by hydroxyproline analysis, which showed the highest HYP content in the Cur/ADP gel group, followed by the curcumin gel group (Figure 5e). Together, these results demonstrate that the Cur/ADP-loaded GelMA hydrogel markedly accelerated wound closure, promoted re-epithelialization, and enhanced collagen deposition, resulting in improved skin regeneration. The enhanced tissue regeneration, as demonstrated by the thicker neo-epidermis, increased collagen deposition, and improved hydroxyproline content in the Cur/ADP hydrogel-treated wounds, also supports the findings of Li et al. and Wang et al., who reported that bioactive GelMA systems enhance collagen formation and tissue remodeling [30].

### 2.6. In Vivo Regulation of Inflammatory and Repair-Related Factors by Cur/ADP GelMA Hydrogels

**Figure 6:**
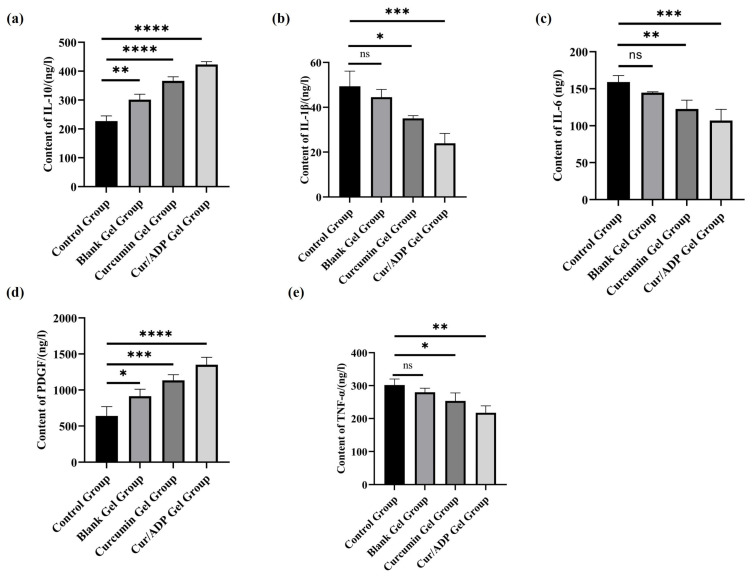
In vivo analysis of anti-inflammatory and pro-inflammatory markers. (**a**) Content of IL-10 in the different treatment groups. (**b**) Content of IL-1β across the treatment groups. (**c**) Content of IL-6 in the various treatment groups. (**d**) Content of PDGF in the different treatment groups. (**e**) Content of TNF-α in the treatment groups. All data are presented as mean ± SD (n = 3). Statistical significance was determined by one-way ANOVA with Tukey’s multiple comparisons test. * *p* < 0.05; ** *p* < 0.01; *** *p* < 0.001; **** *p* < 0.0001; ns = not statistically significant.

The effects of different hydrogel treatments on inflammatory and repair-related cytokines are shown in Figure 6a–e. The anti-inflammatory cytokine IL-10 was markedly increased after hydrogel treatment, with the Cur/ADP gel group showing the highest IL-10 level among all groups, followed by the curcumin gel and blank gel groups (Figure 6a). In contrast, the pro-inflammatory cytokines IL-1β, IL-6, and TNF-α showed a progressive decrease after treatment, and the lowest levels were consistently observed in the Cur/ADP gel group (Figure 6b,c,e)**.** The curcumin gel group also significantly reduced these inflammatory mediators compared with the control and blank gel groups, whereas the blank gel group showed only a limited effect. In addition to suppressing inflammation, hydrogels promoted the expression of the repair-related growth factor PDGF (Figure 6d). The PDGF content gradually increased from the control group to the blank gel, curcumin gel, and Cur/ADP gel groups, with the highest level again detected in the Cur/ADP gel group. These findings indicate that the Cur/ADP-loaded GelMA hydrogel not only attenuated the inflammatory response by downregulating IL-1β, IL-6, and TNF-α while upregulating IL-10, but also enhanced the repair microenvironment through increased PDGF expression. Overall, the dual-drug-loaded hydrogel showed the most pronounced anti-inflammatory and pro-healing effects in vivo. Our in vivo results indicated that the Cur/ADP hydrogel not only inhibited bacterial load but also promoted a favorable inflammatory environment by enhancing anti-inflammatory cytokine (IL-10) levels while reducing pro-inflammatory mediators (IL-1β, IL-6, TNF-α). This is in agreement with previous reports, where curcumin-based materials were shown to reduce inflammation and improve wound healing [31].

### 2.7. In Vivo Revascularization and Biosafety Evaluation of Cur/ADP GelMA Hydrogels

**Figure 7:**
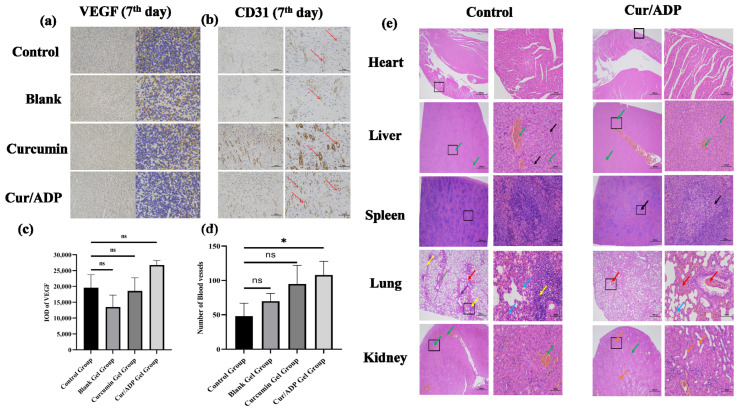
In vivo revascularization capacity of hydrogels. (**a**) Immunohistochemical staining images of VEGF expression on day 7 post-treatment. (**b**) Immunohistochemical staining images of CD31 expression, marking blood vessels, on day 7 post-treatment. (**c**,**d**) Quantitative analysis of the Integrated Optical Density (IOD) of VEGF and CD31, respectively, representing the revascularization capacity. (**e**) Histological evaluation of major organs (heart, liver, spleen, lung, kidney) on day 14 post-implantation, with H&E staining. Representative images show tissue morphology, with color-coded arrows indicating specific features of interest. All data are presented as mean ± SD (n = 3). Statistical significance was determined using one-way ANOVA with Tukey’s multiple comparisons test. * *p* < 0.05; ns = not statistically significant.

The in vivo revascularization capacity of the hydrogels was assessed by immunohistochemical staining of VEGF and CD31 on day 7 (Figure 7a,b). VEGF staining showed a stronger positive signal in the Cur/ADP gel group than in the other groups, while the blank gel group exhibited the weakest expression. The curcumin gel group also showed increased VEGF expression compared with the control and blank gel groups. Quantitative analysis of VEGF IOD followed the same trend, with the highest value observed in the Cur/ADP gel group, although the differences among groups were not statistically significant (Figure 7c). These findings suggest that Cur/ADP treatment promoted VEGF expression and contributed to a more favorable pro-angiogenic microenvironment. Consistent with the VEGF results, CD31 staining revealed a greater number of newly formed blood vessels in the curcumin-containing groups, particularly in the Cur/ADP gel group (Figure 7b). Quantitative analysis confirmed that the number of CD31-positive blood vessels gradually increased from the control group to the blank gel, curcumin gel, and Cur/ADP gel groups. Among them, the Cur/ADP gel group exhibited the highest vessel density and showed a significant increase compared with the control group (Figure 7d). These results indicate that the Cur/ADP-loaded hydrogel more effectively promoted neovascularization during wound healing. The biosafety of the hydrogel was further evaluated by histological examination of major organs, including the heart, liver, spleen, lung, and kidney, on day 14 (Figure 7e). H&E staining showed that the overall tissue architecture of these organs remained intact in both the control and Cur/ADP groups, without obvious pathological abnormalities, inflammatory infiltration, tissue necrosis, or structural damage. These observations demonstrate that hydrogel implantation did not induce apparent systemic toxicity and showed good in vivo biocompatibility. A novel aspect of our study was the enhanced angiogenesis observed in the Cur/ADP hydrogel group, as evidenced by higher levels of VEGF and CD31 positive blood vessels. These results suggest that hydrogel promotes neovascularization, a crucial step in tissue regeneration. Recent research by Lee et al. has demonstrated that curcumin can significantly improve angiogenesis, and similar GelMA-based platforms have been shown to support vascularized repair [32]. The Cur/ADP-loaded GelMA hydrogel further contributes to this process, making it an excellent candidate for treating ischemic wounds and chronic ulcers, which often suffer from impaired blood vessel formation [33,34].

## 3. Conclusions

In this study, a multifunctional Cur/ADP-loaded GelMA hydrogel was successfully developed as a wound dressing with combined hemostatic, antibacterial, anti-inflammatory, pro-angiogenic, and regenerative properties. The hydrogel exhibited a porous and interconnected structure, good swelling capacity, favorable cytocompatibility, rapid sol–gel transition, sustained release of ADP and curcumin, and good tissue adhesion, confirming its suitability for wound-site application. Biologically, the dual-drug-loaded GelMA hydrogel showed superior performance over the blank and single-drug-loaded GelMA hydrogel groups. It effectively reduced blood loss, inhibited the growth of *S. aureus* and *E. coli*, downregulated pro-inflammatory cytokines, increased IL-10 and PDGF levels, promoted VEGF and CD31 expression, enhanced collagen deposition and hydroxyproline content, and accelerated re-epithelialization and wound closure in vivo. Histological evaluation of major organs further confirmed its good biosafety. Overall, the Cur/ADP-loaded GelMA hydrogel provided coordinated support across multiple stages of wound healing, from early hemostasis and infection control to later angiogenesis and tissue remodeling. These findings demonstrate that this Cur/ADP GelMA hydrogel is a promising candidate for effective wound management and skin tissue regeneration. One major limitation is the lack of extensive evaluation of the hydrogel’s performance in chronic wounds and larger animal models, which may limit its current applicability in these clinical settings.

## 4. Materials and Methods

### 4.1. Materials

Gelatin from bovine skin (C_102_H_151_O_39_N_31_), Shanghai Bide Pharmaceutical Technology Co., Ltd., Shanghai, China, CAS: 9000-70-8), methacrylic anhydride (C_8_H_10_O_3_, Shanghai Bide Pharmaceutical Technology Co., Ltd.), sodium tetraborate (CAS: 1330-43-4, Shanghai Bide Pharmaceutical Technology Co., Ltd.), pure curcumin (C_21_H_20_O_6_, ≥95%, Solarbio Life Sciences, Beijing, China, CAS: 458-37-7), adenosine 5′-diphosphate (ADP, ≥98%, Macklin, Shanghai, China), lithium phenyl (2,4,6-trimethylbenzoyl) phosphinate (LAP, C_16_H_16_LiO_3_P, Shanghai Bide Pharmaceutical Technology Co., Ltd.), and hydroxypropyl-β-cyclodextrin (HPβCD, Zibo Qianhui Biotechnology Co., Ltd., Shanghai, China, Batch no. HB240305) were used in the preparation of the hydrogels. Escherichia coli (*E. coli*, ATCC 25922) and Staphylococcus aureus (*S. aureus*, ATCC 6538) were obtained from Nanjing Clinic Biological Technology Co., Ltd., Nanjing, China.

### 4.2. Synthesis Principle and Characterization of GelMA

#### 4.2.1. Synthesis of GelMA

Sodium tetraborate buffer (0.12 mol) was prepared by dissolving in 1192 mL purified water at 50 °C. Gelatin (132 g) was dissolved in the buffer under stirring at 50 °C. Methacrylic anhydride was added in six portions at 0.5 h intervals to achieve the desired substitution degree [35]. After the reaction, the solution was ultrafiltered for 4 h to remove boron. The filtrate was concentrated, then freeze-dried at −80 °C for 36 h to obtain GelMA.

#### 4.2.2. Determination of the Degree of Substitution

The degree of substitution is an important index that affects the properties of GelMA, including its strength and drug release [36]. Two methods were used to determine the degree of substitution in this study [37].

Based on the reduction of free amino groups,

(1) $$DS=(1-\frac{S_{c2}/S_{R2}}{S_{c0}/S_{R0}})\times 100\%$$

DS: degree of substitution; S_c0_: peak area of C peak in gelatin; S_c2_: peak area of C into GelMA.
S_R0_: peak area of R peak in gelatin spectrum; S_R2_: peak area of R peak in GelMA spectrum.

Based on the increase in double bond signals and free amino groups, the calculation formula for DS can be derived [38].

(2) $$DS=\frac{{[S}_{a}+S_{d}-(S_{d0}+S_{a0})]}{S_{c}+{[S}_{a}+S_{d}-(S_{d0}+S_{a0})]}\times 100\%$$

S_a_ and S_d_ represent the integrated areas of methacryloyl vinyl proton signals in GelMA, while S_a0_ and S_d0_ represent the corresponding baseline integrations in native gelatin. S_c_ represents the residual free amino/lysine methylene proton signal after methacryloylation. Thus, the calculated DS indicates the percentage of gelatin amino groups substituted by methacryloyl groups.

### 4.3. Preparation of Hydroxypropyl Beta Cyclodextrin (HPβCD) Curcumin Inclusion Complex

The curcumin-HPβCD inclusion complex was prepared using the co-solvent method. Curcumin (14.7 mg) and HPβCD (116.8 mg) were mixed in ethanol and aqueous solution (1:2 molar ratio). The mixture was stirred at 150 rpm in the dark for 24 h at room temperature, followed by centrifugation and lyophilization [39,40,41].

### 4.4. Preparation and Characterization of the Cur/ADP GelMA Hydrogel

ADP (2%) was incorporated into the final solution after determining its concentration using the Blood Clotting Index method [42]. Curcumin inclusion complex and ADP were added to a 10% *w*/*v* GelMA solution and stirred at 37 °C for 2 h. LAP (0.4% *w*/*v*) was then added and stirred for 1 h. The mixture was exposed to UV light (365 nm) for 30 s to initiate polymerization and stored at 4 °C.

### 4.5. SEM Analysis

The microstructure and pore morphology of hydrogels were observed using SEM (XL 30 ESEM FEC, FEI Company, Hillsboro, OR, USA) after lyophilization, swelling, cross-sectioning, and gold sputter coating [43,44].

### 4.6. Swelling Behavior

The swelling ratio was determined by immersing the lyophilized hydrogel samples in PBS at 37 °C. The weight before (w0) and after swelling (w1) was measured, and the swelling rate was calculated using [45]

(3) $$Swelling\;rate\;\%=\frac{W1-W0}{W0}\times 100\%$$

### 4.7. In Vitro Drug Release Studies

Curcumin and ADP release from GelMA hydrogels was assessed in PBS (phosphate-buffered saline) at 37 °C. The absorbance of release samples was measured at 430 nm (curcumin) and 259 nm (ADP) using UV–Vis spectrometry [46,47,48].

### 4.8. Cell Viability Assay

Cytotoxicity was assessed using the CCK-8 assay on L929 cells. Cells were cultured in 96-well plates and incubated with hydrogel leachates for 24 h. Absorbance at 450 nm was measured to determine cell viability [49,50].

### 4.9. Evaluation of the Antibacterial Properties of the Hydrogel

Antibacterial activity of the hydrogels was evaluated against *S. aureus* and *E. coli.* Hydrogels were incubated with bacterial suspensions, and antibacterial activity was assessed using the plate counting method [51].

### 4.10. Evaluation of the Hemostatic Properties of the Cur/ADP GelMA Hydrogel

#### 4.10.1. Blood Clotting Index

The pre-coagulation activity of hydrogels was measured by mixing anticoagulated blood with a hydrogel sample and determining absorbance at 545 nm. BCI was calculated using [52,53]

(4) $$BCI\;(\%)=\frac{Abs\;sample}{Abs\;blank}\times 100\%$$

#### 4.10.2. In Vivo Hemostatic Test

The hemostatic capability was assessed in SD rats using a tail amputation model. The amount of blood absorbed by the hydrogel was measured by weighing the filter paper before and after application [53,54]: Wc represents the weight of filter paper before absorbing blood and Wa represents the weight of filter paper after absorption.

(5) $$Blood\;loss\;volume\left(g\right)=Wa-Wc$$

### 4.11. Animal Skin Wound Model

#### 4.11.1. Skin Irritation Test

The skin irritation potential was assessed by applying GelMA hydrogel to the dorsal skin of rats for seven consecutive days, and erythema and edema were visually recorded.

#### 4.11.2. Wound Healing Assessment

A skin wound model was created on rats using chloral hydrate and depilatory cream. Rats were treated with blank, curcumin-loaded, ADP-loaded and Cur/ADP-loaded gel, and wound closure was measured at 0, 3, 5, 7, and 14 days using ImageJ software (version 1.53t). The wound closure rate was calculated as [51,55]

(6) $$Wound\;closure\;Rate=\frac{\left(Initial\;Wound\;Area-Current\;Wound\;Area\right)}{Intial\;wound\;Area}\times 100$$

#### 4.11.3. Inflammatory Response and Collagen Content

On day 3, skin tissues were analyzed for inflammatory cytokines (IL-6, IL-10, IL-1β, TNF-α) and collagen content (hydroxyproline, PDGF).

#### 4.11.4. Bacterial Load Detection in Skin Wounds

Bacterial load in the wounds was evaluated by homogenizing tissue samples and plating them on solid media. The bacterial count was expressed as CFU per gram of tissue [56]. The bacterial load was determined using the following formula [51]:

(7) $$Bacterial\;load\;(CFU/g)=\frac{Number\;of\;colonies\left(CFU\right)\times Dilution\;Factor}{Tissue\;Weight\;in\;(g)}$$

#### 4.11.5. Capillary Formation Analysis

CD31 and VEGF expression were measured by immunohistochemistry on day 7 to assess new capillary formation in wound tissues [57].

#### 4.11.6. Pathological Observation of Skin Wounds

Five rats from each group were randomly selected, and skin tissue from the wound site was carefully excised. Blood stains were washed with normal saline, excess saline was wiped off with gauze, and the tissue was fixed in 4% paraformaldehyde. Granulation tissue growth and skin maturation were assessed using hematoxylin and eosin (H&E) staining, while collagen deposition was evaluated by Masson’s trichrome staining [58] on the 14th day of treatment.

## Data Availability

The original contributions presented in the study are included in the article. Further inquiries can be directed to the corresponding author.
